# A protocol for the HEaring Impairment Data Infrastructure (HEIDI) study

**DOI:** 10.1371/journal.pone.0320294

**Published:** 2025-05-07

**Authors:** Yvonne Tran, Mariano Cabezas, Frank Tran, Jo-Anne Manski-Nankervis, Jitendra Jonnagaddala, Diana Tang, Kompal Sinha, Mohammad Nure Alam, Jessica Monaghan, Andrew Donald, Rebecca Mitchell, Matthew Crossley, Niloufer Selvadurai, Bamini Gopinath

**Affiliations:** 1 Macquarie University Hearing Research Centre, Faculty of Medicine, Health and Human Sciences, Macquarie University, Sydney, New South Wales, Australia; 2 Department of General Practice and Primary Care, Faculty of Medicine, Dentistry and Health Sciences, University of Melbourne, Melbourne, Victoria, Australia; 3 School of Population Health, Faculty of Medicine & Health, University of New South Wales, Sydney, New South Wales, Australia; 4 Department of Economics, Macquarie Business School, Macquarie University, Sydney, New South Wales, Australia; 5 National Acoustics Laboratory, Sydney, New South Wales, Australia; 6 Department of Management, Macquarie Business School, Macquarie University, Sydney, New South Wales, Australia; 7 School of Psychological Sciences, Faculty of Medicine & Health, University of New South Wales, Sydney, New South Wales, Australia; 8 Macquarie Law School, Macquarie University, Sydney, New South Wales, Australia; University of Southampton - Highfield Campus: University of Southampton, UNITED KINGDOM OF GREAT BRITAIN AND NORTHERN IRELAND

## Abstract

**Background:**

Research suggests that early detection of hearing loss, coupled with prompt and appropriate treatment, can significantly alleviate its negative impacts. Routinely collected real-world data, such as those from electronic health records data, provide an opportunity to enhance our understanding of the management of hearing loss. This project aims to create the HEaring Impairment Data Infrastructure (HEIDI) data lake by assembling datasets from general practice (GP), audiology clinic registries, and cohort studies to investigate hearing-impaired patients’ care pathways. This study seeks to answer key research questions such as “How do patients with hearing loss navigate the care pathway from general practice clinics to audiology clinics?”.

**Methods and analysis:**

The HEIDI data lake will be hosted in a secure research environment at Macquarie University, Sydney, Australia, that complies with Australian legal and ethical requirements to protect patient privacy. Afterwards, new integrated datasets will be built through data linkage of hearing and GP datasets. Finally, the HEIDI data warehouse will be developed and used as a stand-alone dataset for future research. Descriptive and predictive analytics will be undertaken to answer our research questions with the data warehouse. Descriptive analysis will include both conventional and advanced statistical techniques and visualisation that will help us understand the journey of patients with hearing loss. Machine learning strategies such as deep neural networks, support vector machines, and random forests for predictive analytics will also be employed to identify participants that could benefit from proactive management by their GP and determine the effect of interventions through the patient’s journey (e.g., referrals to specialist) on outcomes (e.g., adherence to the intervention).

**Dissemination:**

The findings will be disseminated widely through academic journals, conferences and other presentations.

## 1. Introduction

Hearing loss is one of the most prevalent chronic conditions and is in the top three leading causes of disability [1]. When not addressed, adult-onset hearing loss negatively impacts participation in social activities, mental cognitive and economic health, functional independence, ageing, and overall lifespan [2–5]. This highlights that hearing loss is not isolated; it involves multiple factors, including co-morbid health conditions, socioeconomic circumstances, and lifestyle [6–8]. Studies have indicated that early identification, followed by seeking timely and appropriate treatment and intervention, will significantly alleviate the adverse effects attributable to hearing loss. These proactive measures can promote independent living in later life and better overall well-being [9,10]. Despite these potential benefits, diagnosing, treating, and managing hearing loss is still complex and daunting [11]. While historically, there has not been a clear pathway to navigate these complexities, a pathway covering six distinct stages corresponding to the help-seeking process for addressing hearing loss has been suggested. These stages are (1) Self-diagnosis/Screening (2) Initial contact with a health provider (3) Referral to a hearing health care professional (4) Hearing device recommendation (5) Compliance with the recommendation to obtain a hearing device and (6) Adherence with the recommendation to use a hearing device [12,13].

The process of going from the initial self-diagnosis/screening to adherence using hearing devices, involves multiple steps, each with its challenges. The complexity of this process stems from the combination of medical intricacies associated with hearing loss and the social, psychological and financial factors that patients encounter at each stage [14]. This requires collaboration among audiologists and primary care providers and a broad understanding of the various factors contributing to hearing impairment. To enhance the understanding of the management of hearing loss, make informed decisions and tailor interventions, a data-centric approach is required [15]. Developing a data lake that holistically captures hearing-related information can be highly beneficial and help healthcare providers comprehend their patients better. Without such data, the healthcare system risks operating in silos, failing to address the root causes of barriers in the care pathway [16].

To improve hearing healthcare, this project involves the creation of a *data lake*- a centralised repository of high-quality raw datasets pooled from large, specialised datasets (including participants with and without hearing loss). These datasets will be analysed, to evaluate current patterns of screening and management of adult-onset hearing loss, providing an inclusive view of the journey of hearing-impaired patients. Through this project, the study aims to provide a cross-disciplinary understanding of hearing health within the broader landscape of primary care evaluations. Specifically, the study aims to:

1. Create the HEaring Impairment Data Infrastructure (HEIDI) by assembling and collating datasets from general practice, audiology clinic registries, and cohort studies to provide information on the hearing-impaired patients’ journey.
2. Build unique datasets by linking the general practice datasets and the audiology clinical dataset. These unique datasets will provide information on sociodemographic, clinical observations, diagnostics, therapeutics, audiological measures, and intervention types.
3. Develop a structured and filtered data warehouse from the data within the data lake, to generate evidence that will inform the design of more sensitive tools that can be used broadly to identify people with hearing loss who are more likely to benefit from hearing intervention and aural rehabilitation.
4. Systematically examine how patients with hearing loss navigate the care pathway from general practice clinics to audiology clinics.

## 2. Methods

**Fig 1:**
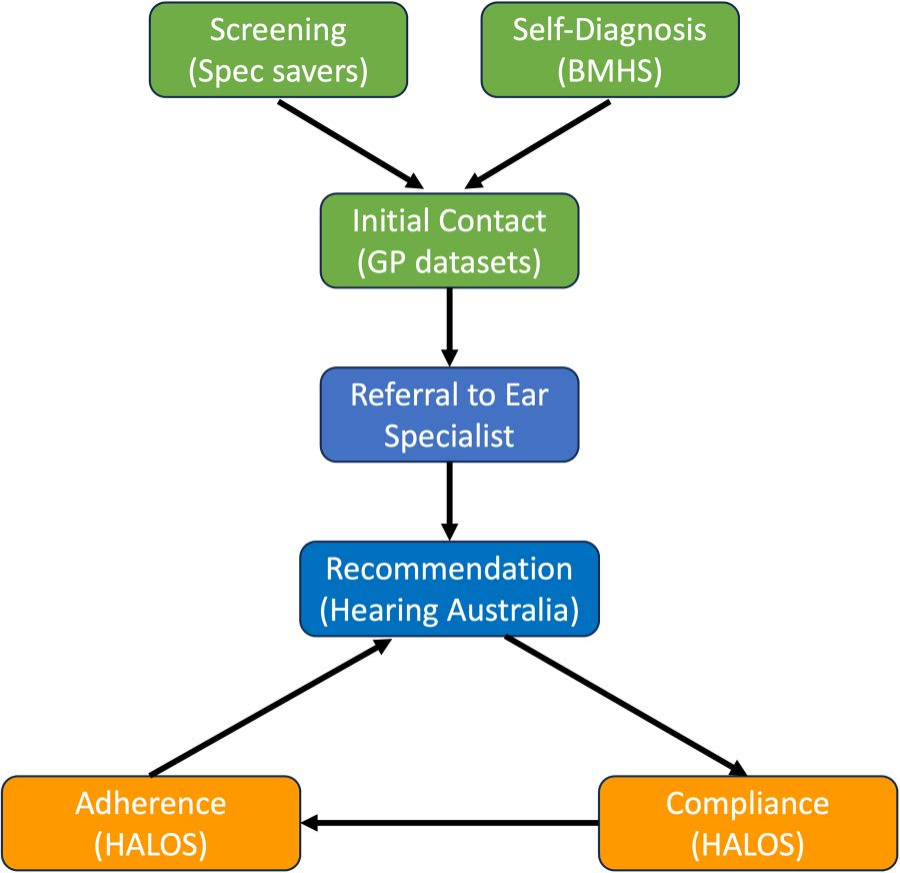
Data lake scheme based on the help-seeking pathway introduced by Benova et al. [ **13].** The nodes are coloured according to the stages with green nodes for (1) Self-diagnosis/Screening and (2) Initial contact with a health provider, blue nodes for (3) Referral to a hearing health care professional and (4) Hearing device recommendation and orange nodes for (5) Compliance with the recommendation to obtain a hearing device and (6) Adherence with the recommendation to use a hearing device.

This study was approved by the Macquarie University Human Research Ethics Committee (ID: 11790) on 22^nd^ of February 2023. The HEIDI study has a data infrastructure and data linkage research design (see timeline in Table 1). The data infrastructure will be in the format of a secure virtual machine (VM) that sits within the secure servers of Macquarie University and has restricted access to members of the research team, as required by the legal agreements with the custodians of the original raw datasets. Data linkage will then occur between the hearing datasets and the GP datasets to allow for a flow of information between early stages such as “Initial Contact with a Health Provider” and later stages such as the “Hearing Device Recommendation” stages (See Fig 1). The project will involve the aggregation of existing data and focuses on the setup of novel data infrastructure that will allow secondary data analysis and data analytics on anonymised or de-identified data.

**Table 1:** Milestones and study timeline.

Milestone	Due date
Submission of the ethics application to Macquarie University	13^th^ February 2023
Project staff hiring and establishment of the project advisory group	25^th^ March 2023
Start of the data pooling from different sources to create the data lake	16^th^ January 2024
Creation of the data warehouse through data linkage, cleansing and standardisation	19^th^ August 2024
Descriptive statistics on patient journeys	29^th^ September 2024
Development of predictive models using machine learning	4^th^ November 2024
Dissemination of project findings (e.g., reports, publications, conference/ meeting presentations).	21^st^ February 2025

### 2.1 Datasets

The datasets for this study can be integrated into the help-seeking pathway introduced by Benova and colleagues (seen in Fig 1) [13]. There are three main areas for the datasets — cohort studies, hearing-related and general practice — giving the study a multidisciplinary perspective. A comprehensive understanding of factors that influence hearing loss from sociodemographic and medical to lifestyle-related aspects will be obtained. Informed consent to share the data was obtained independently by the data custodians for all the participants of each study. A summary of the existing datasets and the types of data collected, to be included in the data lake can be found in Table 2.

**Table 2:** Summary of Australian datasets and data registries to be included in the data lake.

Dataset source (data collection years)		Description	Individuals (N) [Age range]	Type of data collected
Research datasets	BMHS (1997–2009)	Landmark sensory loss (vision and hearing) study in Western Sydney focusing on older adults.	2,956 [54–99]	Socio-demographic; medical history; objective/self-reported hearing loss; help-seeking for hearing loss; clinician visits; treatment received for hearing (e.g., GP, otorhinolaryngologist, audiologist); use of hearing devices
	HALOS (2021 –)	Mixed methods study on health and social outcomes in adults using a hearing device.	908 [40–96]	
Hearing health services datasets	Hearing Australia (2016–2023)	Deidentified client data from the nation’s largest provider of government-funded hearing services	~90,000 [32–108]	Treatment received for hearing (e.g., GP, otorhinolaryngologist, audiologist); use of hearing devices; audiometric measures
	SpecSavers (2015–2023)	Anonymised hearing screening data (audiometry) from a private company providing hearing care in Australia and New Zealand.	~2,600,000 [18–124]	Demographics (year of birth); audiometric features
General practice datasets	Patron (2016–2023)	Deidentified patient data from ~117 general practices, mainly from the state of Victoria.	~800,000 [34–132]	observations; family history; medical history; non-clinical encounter characteristics; encounter reason; investigations requested; Medicare Benefits Schedule (MBS) billing item.
	ePBRN (2009–2019)	Deidentified patient data from general practices in Southwest Sydney.	~250,000 [0–120]	

#### 2.1.1 Cohort study datasets.

**The Blue Mountains Hearing Study (BMHS):** The BMHS is a sub-study from the Blue Mountains Cohort Study, which examined the long-term development and progression of sensory problems such as vision and hearing loss. From 1997 to 2009, 2956 persons 50 years or older had audiometric testing performed in addition to the health outcomes measures from the Blue Mountains Cohort study. The BMHS contains de-identified sociodemographic, lifestyle factors, health outcomes and audiometric data from 1997 to 2009. This is a population-based survey of older adults from the Blue Mountains area, west of Sydney, Australia [17].

**Hearing impairment in adults: A Longitudinal Outcomes Study (HALOS):** HALOS is a mixed-methods study collecting both cross-sectional and longitudinal data on health and social outcomes from hearing aids and cochlear implant users [18]. It is currently undergoing the first wave of data collection. The quantitative component will collect audiological, health, psychosocial, functional and employment outcomes using validated instruments. The qualitative component will consist of semi-structured interviews aimed at understanding the patient journey and perspectives on the Australian hearing service model.

#### 2.1.2 Hearing-related health services datasets.

Hearing related datasets will be drawn from two sources: Hearing Australia and Specsavers Australia and New Zealand.

**Hearing Australia:** The Hearing Australia dataset is a clinical registry from Australia’s largest provider of government-funded hearing services, e.g., pensioners, and veterans (access to ~ 90,000 clients). Information on the types of interventions provided along with tracking of individuals’ hearing health over time where applicable will be obtained. This data will be linked with the GP datasets.

**Specsavers Australia and New Zealand:** Specsavers is an international private company that specialises in eye and hearing care. Currently, they have multiple locations throughout Australia and New Zealand and, in 2017, they introduced audiology as one of their services. Since then, over 2 million audiology tests have been performed on adults aged 40 + . This data set contains basic demographic and audiometric measurement data, which will be used for the study. This effort aligns with their mission to make high-quality hearing care accessible to a broader audience. While this data will be useful to understand general hearing patterns in Australia and New Zealand, linkage with other services will not be possible due to the data anonymisation process.

#### 2.1.3 General practice datasets.

**Patron**: The Patron primary care repository contains deidentified data from general practice primarily in Victoria, Australia (interstate practices can also participate) [19,20]. It is part of the Data for Decisions research initiative at the Department of General Practice and Primary Care at the University of Melbourne. The dataset aims to facilitate research that will improve patient care in general practice and benefit the community overall. Patron is a rich repository of de-identified general practice clinical data that can be linked to other datasets (in our case, the Hearing Australia dataset). This provides a unique opportunity to address a wide range of research and policy questions.

**Electronic Practice Based Research Network (ePBRN)**: The ePBRN dataset is a large and diverse collection of health-related information obtained from electronic health records (EHRs) [21,22]. The data repository consists of pseudonymised patients from a network of electronic health records of general practices in an integrated health neighbourhood in Southwest Sydney. The dataset also contains a rich and diverse range of data elements, which enables researchers to explore complex relationships between different health factors and outcomes.

### 2.2 Creating the HEIDI data lake

The data lake will be created within a VM that sits on the servers of Macquarie University. The VM will run on the VMWare Virtual platform using the Windows 11 Pro (64 bits) operating system. In terms of hardware, the virtual platform will comprise 2 Intel® Xeon® Gold 6238R CPU processors at 2.20 GHz, 32 gigabytes of RAM, 1 terabyte of hard drive space. As a virtual platform with encrypted access, it is a highly secure research environment that is compliant with requirements of protecting patient information for medical data (2-factor authentication with password protected accounts and requiring VPN access). Furthermore, the technology being used is commonly used for projects that deal with aggregated data and addresses security concerns regarding data access [23–25].

The datasets will be received from the data custodians securely and, either automated data imports from these data sources will be remotely and securely performed or the data will be moved through a secure encrypted removable hard drive with a decryption key provided separately. Using a secure and private VM cloud hosting environment from Macquarie University servers, data remains in the control of the data custodians of the datasets and there will be no need for any sharing of identified raw data. Furthermore, all data will be imported in an anonymised or de-identified format.

The datasets will be uploaded into the data lake (as part of the VM) and imported to the Structure Query Language (SQL) Server 2022 database from Microsoft. Using SQL Server Management Studio v19.1, each data source will be stored and managed as its own database through SQL queries. The uploading method into SQL Server will vary based on the format of the data source, however, each dataset will be downloaded directly into the VM, and any pre-processing or transformation of the data will only occur inside the VM through SQL queries, to ensure the security and privacy of the data.

### 2.3 Data linkage

To understand the help-seeking pathway, the Hearing Australia database will be linked to the GP datasets. Data linkage will be conducted using the Generic Health Network Information Technology for the Enterprise (GRHANITE^™^) data extraction tool and will be conducted within the data custodian’s site. GRHANITE^TM^ will be used to generate encrypted keys that can be used to record-link data between two or more independent databases without exporting patient identifiers (e.g., name, date of birth, postcode etc). The encryption used is non-reversible, meaning there is no way for the identity of an individual to be determined from the information used in the record linkage process. Once the datasets have encrypted keys, the de-identified data will be imported into the HEIDI data lake. Data linkages will then be conducted within the secure research environment and matching the encrypted keys between the independent datasets. Through linkage of both GP events (visits, medication, other diagnoses, etc.) and hearing-related events (audiometry tests and specialist visits), the patient journeys will be established.

A previous study from Australia, the Bettering the Evaluation and Care of Health (BEACH) study, showed that only about 3 per 1000 GP consultations with patients aged 50 years involved management of age-related hearing loss. Taking that statistic into account and the fact that hearing-related datasets would only include people with hearing loss, we expect to detect a similar percentage of patient with adult-onset hearing loss (0.3%) through filtering [26].

### 2.4 HEIDI data warehouse

To create a structured, filtered data warehouse, cleansed data from the HEIDI data lake will be used. As the different datasets are heterogeneous, all datasets will be examined individually to determine the set of variables that are common (e.g., age, gender, visit reason, etc.). From this examination, two types of new SQL tables will be created: a general patient table with demographics and immutable data, and event tables describing different possible events through the patient journeys (e.g., visit with a GP, audiometry tests, diagnostics, medication, etc.). For each type of event, common variables among datasets will be pooled together (e.g., visit reason). Considering that Patron already has a standardised format for three different EHR providers (Best Practice, Medical Director and Zedmed) and that ePBRN aggregates data from two EHR providers with mostly the original format (Best Practice and Medical Director), we will follow a structure that is similar to Patron when creating the data warehouse.

The values of each variable will be standardised across datasets for consistency and will allow for the consolidation of data from different data sources. This process will involve conversion to common SQL datatypes depending on the variable types (e.g., conversion to the same numerical datatype for numerical variables) and scaling as necessary. For variables with more than 50% missing values, the whole variable will be dropped when building the data warehouse. For variables with less than 50% missing values, data imputation techniques will be used according to the variable type (e.g., mean/ median imputation for numerical variables and frequent category imputation for categorical variables). Similarly, row entries on the databases with more than 50% missing values will be dropped during the creation of the warehouse.

Once the data is cleansed and standardised, the whole patient journey will be tracked and specific variables that capture information relevant to adult-onset hearing loss will be created. Several filtering techniques will be used to achieve the latter, including filters for:

1. Medical codes (including Medicare Benefits Schedule item numbers) associated with hearing loss encoded on the raw datasets [17].
2. The age of the patient when they were screened or diagnosed with a hearing loss.
3. String/Substring matching for key words related to the hearing loss pathway, such as ‘audiologist’ and ‘ENT’ for referrals, ‘deafness’ and ‘hearing loss’ for visit reasons or diagnoses and use of certain medications which are known hearing loss risks factors, e.g., chemotherapy drugs [27].

The number of different datasets generated for the data warehouse will be determined by the diverse range of queries posed. These queries will be curated to explore the relationship between patient profiles and their outcomes in the different stages of the help-seeking process [12]. Through these queries, insightful views of the consolidated data within the warehouse which can be used as stand-alone datasets for future queries will be generated. For this project, the data warehouse will be built such that it is structured optimally to support downstream tasks associated with patient-centred health care for hearing loss.

There are three main categories of questions and views of data to explore within the HEIDI data warehouse, as represented in Fig 1; Screening/Self-diagnosis, Intervention and Post-Intervention.

#### 2.4.1 Screening/Self-diagnosis queries.

The earliest stage in the help-seeking process is the screening or self-diagnosis stage. This is captured in our datasets via screening data provided by Specsavers, and “Reason for Visit” and “Medical History” provided by our GP datasets.

Some queries which will provide insight into this stage include:

1. Of those with adult-onset hearing loss, at what age did they have a screening test performed?
2. At what age did they visit their GP about their hearing?
3. Is there a relationship between the severity of hearing loss and the date of their first screening or first GP visit related to hearing?

#### 2.4.2 Intervention queries.

Intervention will be defined as the various actions that are taken by the patient and/or the GP to manage hearing loss in the patient, after the initial contact. These include referrals to audiologists and Ear, Nose and Throat surgeons (ENTs), performing hearing screenings, as well as any recommended care plans provided by the GP. Relevant intervention-related queries include:

1. What were the first interventions provided by the GP in response to a hearing-loss diagnosis?
2. Do care plans or intervention procedures exist for patients with hearing loss?
3. Are the interventions standardised across GP clinics?

#### 2.4.3 Post-intervention outcomes.

Analysing the outcomes of the intervention will allow us to evaluate the effectiveness of the intervention. Post-intervention can also refer to the consequent interventions suggested for the patient as well as the monitoring of their adherence and compliance to the intervention. Examples of queries related to post-intervention include:

1. After a referral to an audiologist/ENT, did the patient have any further hearing-related visits to the GP?
2. What aspects of the patient data changed after accessing hearing intervention(s)?
3. What post-intervention data quantifies a good outcome?

### 2.5 Statistical analysis

Once the HEIDI data lake and data warehouse are established, provision and analysis of data can be conducted within the secure VM. Data analytics will be performed using an in-browser analytics platform with a streamlined user experience and interoperability with advanced analytic tools such as SAS, R and Python. This will involve both descriptive and predictive models. This dual approach can enhance patient care, has the potential to optimise resource allocation, and may contribute to better overall health outcomes in the field of audiology.

Descriptive analytics – automation tools that group, screen, compare and summarise data to generate evidence, which will provide insights to what is existing and already occurring in the general practice setting regarding hearing loss, will be created. Analysis conducted includes conventional statistical methods such as descriptive statistics, regression modelling, comparison testing (ANOVA, linear mixed models) etc. Data visualisation will also be conducted.

Predictive analytics - models to identify patients with hearing loss who would benefit from proactive care and management from GPs will be built using machine learning strategies such as deep neural networks, support vector models, random forest, and logistic modelling.

## 3. Discussion

Hearing loss as a medical condition is complex, it is known that when unaddressed, it can significantly impair communication abilities, social participation, mental and cognitive health, functional independence and impose economic burdens [2–5]. The diverse impacts of hearing loss highlight the urgency of developing comprehensive strategies for its management and treatment. To enhance our understanding and management of hearing loss developing a data-centric approach is imperative. This involves harnessing multiple data sources, creating unique datasets, and utilising advanced analytics. Given the complexities of the problem, this necessitates the integration of diverse data sources that cover medical attributes and lifestyle factors. Creating unique datasets that link these varied sources enables a more holistic view of the patient and the condition. Also, by mapping the help-seeking pathway, the patient journey for individuals with hearing loss can be better understood.

In this unique project, analytical methods, including machine learning and artificial intelligence, will be used to identify patterns and trends that are not immediately apparent, leading to a better understanding of patient journeys and the progression and management of hearing loss. For example, by leveraging machine learning techniques, invaluable insights for tailoring interventions and improving access to care can be identified [28]. By employing these methods, the study aims to extract and communicate valuable insights from data, providing a solid foundation for informed decision-making and strategy development in the context of hearing loss management, particularly within the general practice setting. Consequently, this study will contribute significantly to the understanding of hearing loss and outcomes from the study can be used by policymakers, healthcare providers, and advocacy groups. By bridging the gap between data and real-world application, this study hopes to enhance the entire spectrum of hearing loss management, from early detection to effective treatment and beyond.
